# Organoselenium Compounds as Novel Adjuvants of Chemotherapy Drugs—A Promising Approach to Fight Cancer Drug Resistance

**DOI:** 10.3390/molecules24020336

**Published:** 2019-01-18

**Authors:** Gabriella Spengler, Márió Gajdács, Małgorzata Anna Marć, Enrique Domínguez-Álvarez, Carmen Sanmartín

**Affiliations:** 1 Department of Medical Microbiology and Immunobiology, Faculty of Medicine, University of Szeged, Dóm tér 10, 6720 Szeged, Hungary; spengler.gabriella@med.u-szeged.hu (G.S.); mariopharma92@gmail.com (M.G.); 2 Interdisciplinary Excellence Centre, Department of Inorganic and Analytical Chemistry, University of Szeged, Dóm tér 7, 6720 Szeged, Hungary; marcmalgorzata@gmail.com; 3 Instituto de Química Orgánica General, Consejo Superior de Investigaciones Científicas (IQOG-CSIC), Juan de la Cierva 3, 28006 Madrid, Spain; 4 Department of Pharmaceutical Technology and Chemistry, School of Pharmacy and Nutrition, University of Navarra, 31008 Pamplona, Spain; 5 Instituto de Investigaciones Sanitarias de Navarra (IdiSNA), 31008 Pamplona, Spain

**Keywords:** anticancer, combination, checkerboard, selenium, lymphoma, doxorubicin, topotecan

## Abstract

Malignant diseases present a serious public health burden and their treatment with traditional chemotherapy cannot be considered an all-round solution, due to toxic side effects. Selenium compounds (Se-compounds) have received substantial attention in medicinal chemistry, especially in experimental chemotherapy, both as cytotoxic agents and adjuvants in chemotherapy. A checkerboard microplate method was applied to study the drug interactions of Se-compounds and clinically relevant chemotherapeutic drugs against the multidrug-resistant (MDR) subtype of mouse t-lymphoma cells overexpressing the ABCB1 transporter. Se-compounds showed synergistic interactions with chemotherapeutic agents targeting the topoisomerase enzymes or the microtubule apparatus. The ketone-containing selenoesters showed synergism at lower concentrations (1.25 µM). Most of the tested compounds interacted antagonistically with alkylating agents and verapamil. A thiophene-containing Se-compound showed synergism with all tested drugs, except cisplatin. While the exact mechanism of drug interactions is yet unknown, the potency of the selenocompounds as efflux pump inhibitors or the potentiation of their efficacy as reactive oxygen species modulators may play a role in their complementary activity against the tested MDR lymphoma cell line.

## 1. Introduction

Malignant diseases are a significant public health burden, accountable for one-sixth of deaths globally and have an estimated total economic cost of 1 trillion United States (US) dollars [1]. Lymphomas are blood cancers, originating from the body’s own immune cells (lymphocytes), affecting around 111,000 people (Hodgkin-lymphomas: ~18,000; non-Hodgkin-lymphomas: ~93,000) in the European Union (EU) alone (2012) [2]. High grade/rapidly growing lymphomas (frequently affecting children) are very often difficult to treat [3,4,5,6,7]. Traditional (cytotoxic) chemotherapy is still the first-line treatment for an overwhelming majority of tumors, together with radiation and surgical intervention [8,9,10]. Nevertheless, chemotherapy is coupled with serious, clinically significant side effects, some that are foreseeable and characteristic for all such agents (due to their effect on rapidly dividing normal cells in the body), while others are specific to some drugs (e.g., cardiomyopathy related to doxorubicin, hemorrhagic cystitis related to acrolein, a toxic metabolite of cyclophosphamide), frequently leading to treatment discontinuation and decreased quality of life (QoL) for the patients [11,12,13,14,15]. The importance and success of combination chemotherapy in the treatment of malignant diseases has been described in detail both in the laboratory setting as well as in clinical practice [16,17]. There are numerous studies demonstrating that using multiple chemotherapeutic drugs, having synergistic interactions increases patient survival rate. In addition, this therapeutic strategy allows for the dose reduction of individual drugs [18]. The relevance of combination chemotherapy is further highlighted by the growing clinical problem of cancer multidrug resistance (MDR) and tumor cell heterogeneity, often leading to treatment failure, especially when these drugs are used as monotherapy [13,19,20,21,22,23]. Organoselenium compounds have received substantial attention in medicinal chemistry, due to their pronounced biological and redox-modulating activities, to such a great degree that “bio-selenium research” is being conducted related to cardiovascular, autoimmune, endocrine, neurodegenerative and psychological conditions, from in vitro experiments all the way to clinical trials [24,25,26,27,28]. There are a plethora of studies demonstrating the efficacy of structurally dissimilar selenocompounds as novel anticancer agents, suggesting the significant role of the selenium atom in these molecules [27,29,30,31,32]. The potential of redox-modulating compounds in the management of therapy-refractory lymphomas has also been described [33,34]. In addition, synergistic interactions have been observed between selenium (both in its elemental forms and as various compounds) and a diverse range of chemotherapeutic drugs (cisplatin, irinotecan, imatinib, paclitaxel) on colorectal, breast, lung, and leukemia cell lines, respectively [35,36,37,38,39,40]. 

**Figure 1:**
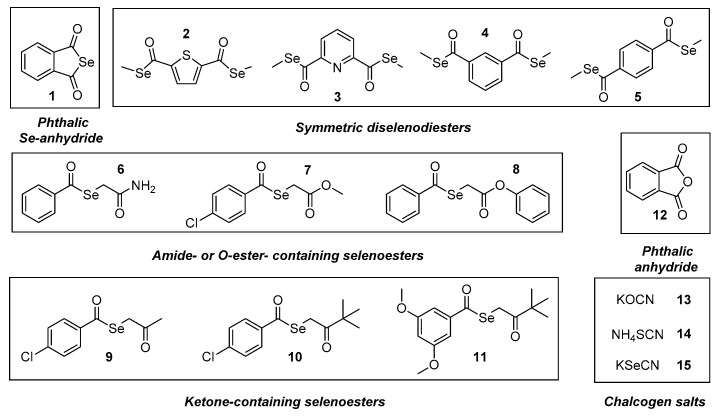
Structure of the tested compounds **1**–**15**.

In our previous studies, the design, synthesis and preliminary biological screening of a cyclic selenoanhydride (**1**) and ten selenoesters (**2**–**11**, Figure 1) were performed [41]. The most active compounds presented very promising anticancer properties on a variety of cell lines (with IC_50_ values in the nanomolar range) and proved to be effective modulators of programmed cell death and of the ABCB1 (ATP-binding cassette subfamily B member 1 or P-glycoprotein) multidrug efflux pump on both murine and human model systems [42,43,44], in addition to having promising absorption, distribution, metabolism, and excretion (ADME) properties based on in silico methods [45]. Based on our previous results, the aim of the current study was to evaluate the potential pharmacological interactions between clinically relevant anticancer drugs in vitro and these compounds with pronounced anticancer activity against lymphoma and to explore the potential applications of these derivatives as co-adjuvants of drugs currently used in cancer chemotherapy. 

## 2. Results and Discussion

In the present study, we have evaluated the abovementioned 11 selenocompounds (Se-compounds), including the selenoanhydride **1** and the selenoesters **2**–**11** (Figure 1). Besides, we have also included the phthalic anhydride (**12**) in our experiments to ascertain the crucial role of the selenium atom in the activity of the phthalic selenoanhydride **1** (**12** is its oxygen isostere), as well as 3 chalcogen XCN salts (compounds **13**–**15**, X=O, S, Se) to compare the activity of the organic selenocompounds **1**–**11** with selected inorganic related salts. This study had a dual purpose: firstly, to determine the efficacy of selenocompounds **1**–**11** as adjuvants in combinational chemotherapy in an in vitro resistant lymphoma model (comparing it with the activity of the compounds **12**–**15**); additionally, to shed a light on the mechanism of action of the tested compounds, because their interactions with the chemotherapeutic agents should be related to their own mechanism of activity. Seven anticancer drugs (Table S1, in Supplementary Material) were tested with different mechanisms of action, to establish the interactions of the respective drugs (one for each mechanism of action) with the different selenocompounds. Briefly, we have evaluated the Se-compounds in combination with two topoisomerase inhibitors (topotecan [*Top*] and doxorubicin [*Dox*]), a microtubule formation inhibitor (vincristine [*Vin*]), two alkylating agents (cisplatin [*Cis*] and cyclophosphamide [*Cpm*]), and two antimetabolites (methotrexate [*Met*] and 5-fluorouracil [*5-FU*]). Besides, we have tested an efflux pump inhibitor (verapamil [*Ver*], which is not an anticancer drug) to evaluate the interaction of the Se-compounds with this alternative mechanism of action. The concentrations at which anticancer drugs and Se-compounds were tested are given in Tables S1 and S2 in the Supplementary Material.

The checkerboard combination assay is a widely used and convenient in vitro method for the assessment of drug interactions among various pharmacological agents, especially when the data obtained are analyzed using CompuSyn software. This program, besides enabling the calculation of the combination indices, also allows the determination of the most effective ratios of combinational agents, which could be relevant for possible subsequent clinical testing [46,47,48]. These combination indexes are used to determine the type of interaction, according to Table 1. 

**Figure 2:**
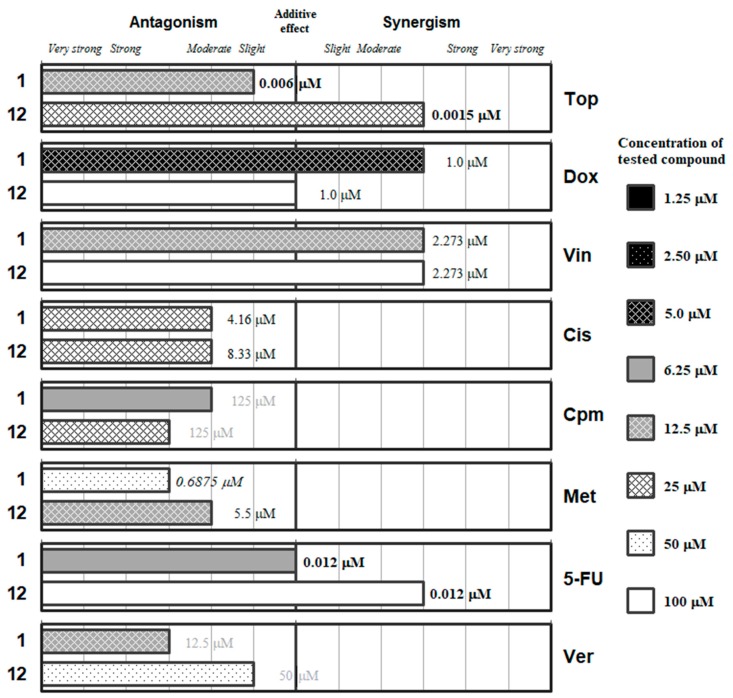
Interactions of the phthalic selenoanhydride (**1**) and the phthalic anhydride (**12**) with a panel of anticancer drugs (topotecan [*Top*], doxorubicin [*Dox*]), vincristine [*Vin*]), cisplatin [*Cis*], cyclophosphamide [*Cpm*]), methotrexate [*Met*] and 5-fluorouracil [5-*FU*]) or an efflux pump inhibitor (Verapamil [*Ver*]). Concentration of the tested compound is given according the legend; concentration of the anticancer drug is given in numbers inside the graph (in bold: below 0.1 µM, in italics between 0.1 µM and 1 µM, and in grey above 10 µM).

**Figure 3:**
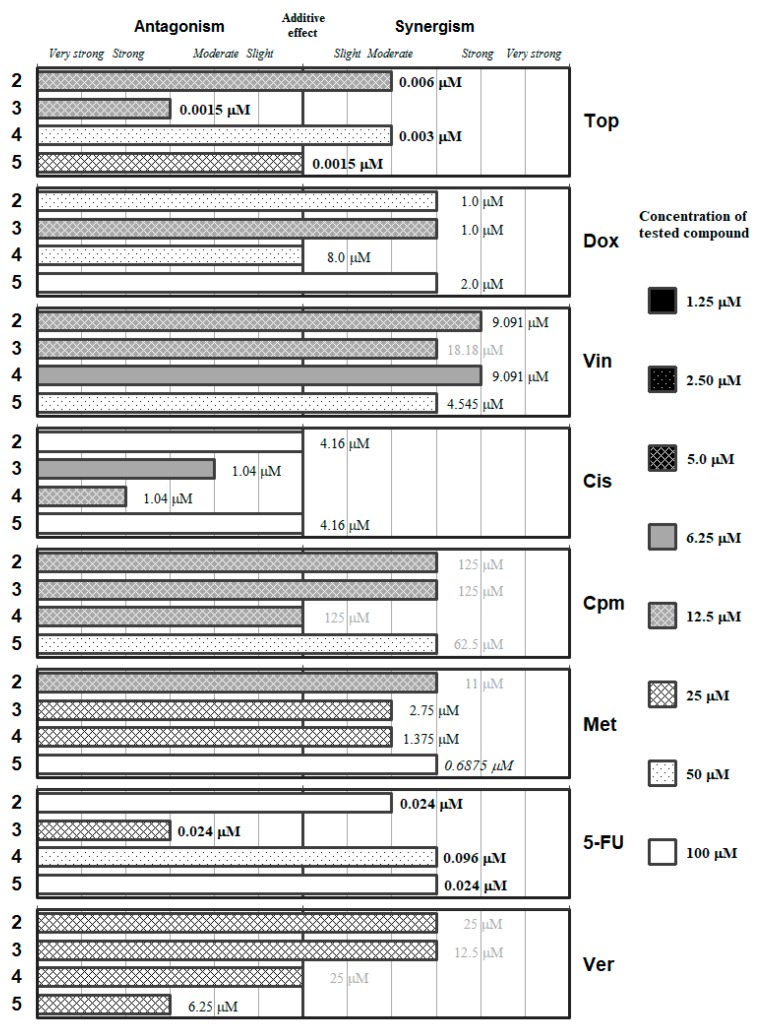
Interactions of the symmetric diselenodiesters (**2**–**5**) with a panel of anticancer drugs (*Top*, *Dox*, *Vin*, *Cis*, *Cpm*, *Met* and 5-*FU*) or an efflux pump inhibitor (*Ver*). Concentration of the tested compound is given according the legend; concentration of the anticancer drug is given in numbers inside the graph (in bold: below 0.1 µM, in italics between 0.1 µM and 1 µM, and in grey above 10 µM).

**Figure 4:**
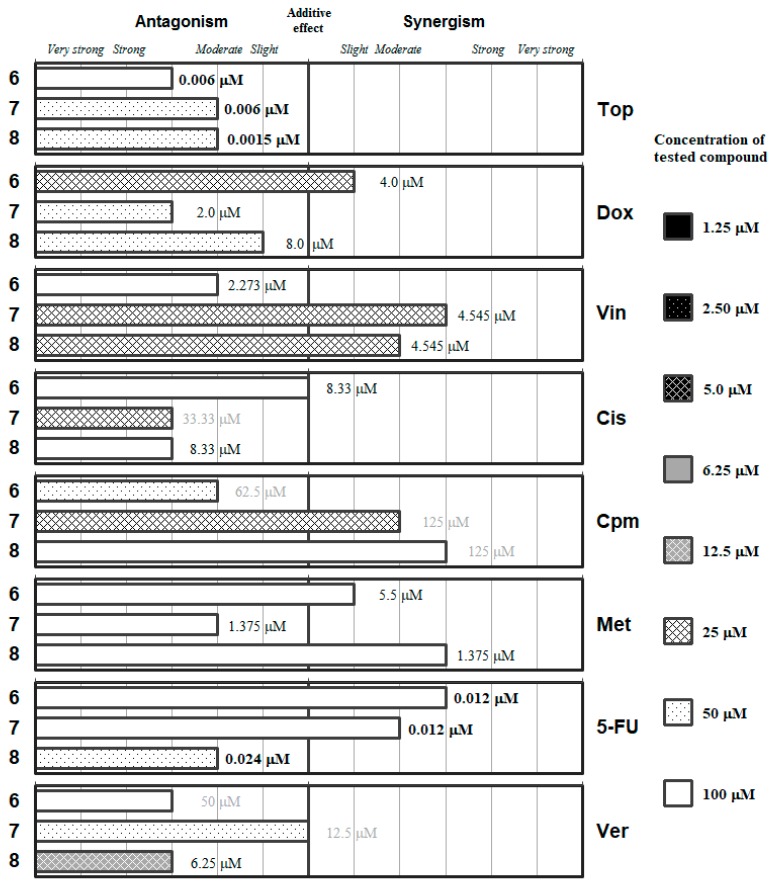
Interactions of the carbamoyl selenoester (**6**) and of the R-ylcarbonyl selenoesters (**7** and **8**) with a panel of anticancer drugs (*Top*, *Dox*, *Vin*, *Cis*, *Cpm*, *Met* and 5-*FU*) or an efflux pump inhibitor (*Ver*). Concentration of the tested compound is given according the legend; concentration of the anticancer drug is given in numbers inside the graph (in bold: below 0.1 µM, in italics between 0.1 µM and 1 µM, and in grey above 10 µM).

**Figure 5:**
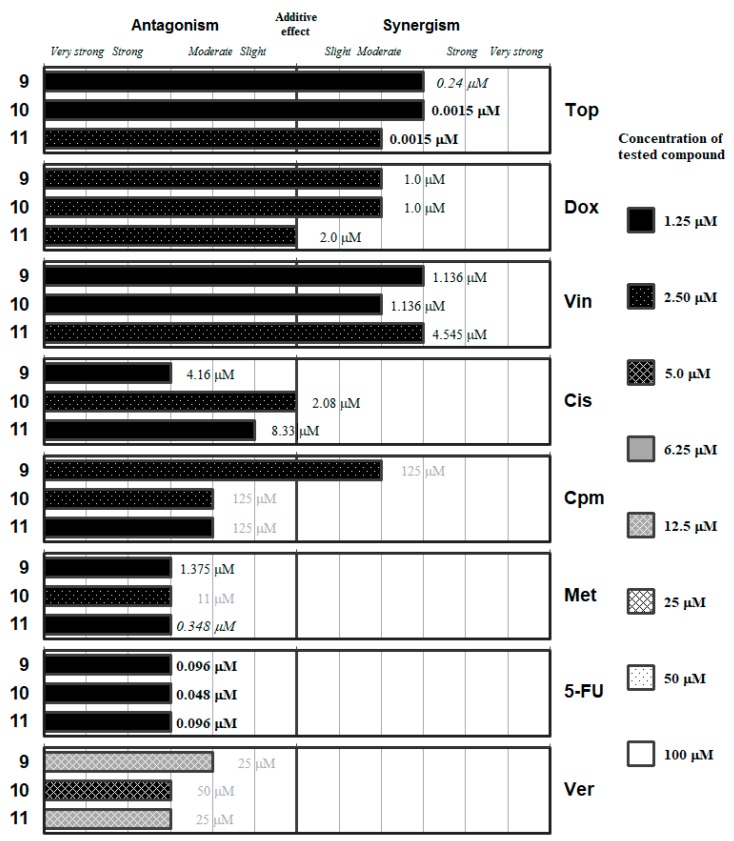
Interactions of the ketone-containing selenoesters (**9**–**11**) with a panel of anticancer drugs (*Top*, *Dox*, *Vin*, *Cis*, *Cpm*, *Met* and 5-*FU*) or an efflux pump inhibitor (*Ver*). Concentration of the tested compound is given according the legend; concentration of the anticancer drug is given in numbers inside the graph (in bold: below 0.1 µM, in italics between 0.1 µM and 1 µM, and in grey above 10 µM).

**Figure 6:**
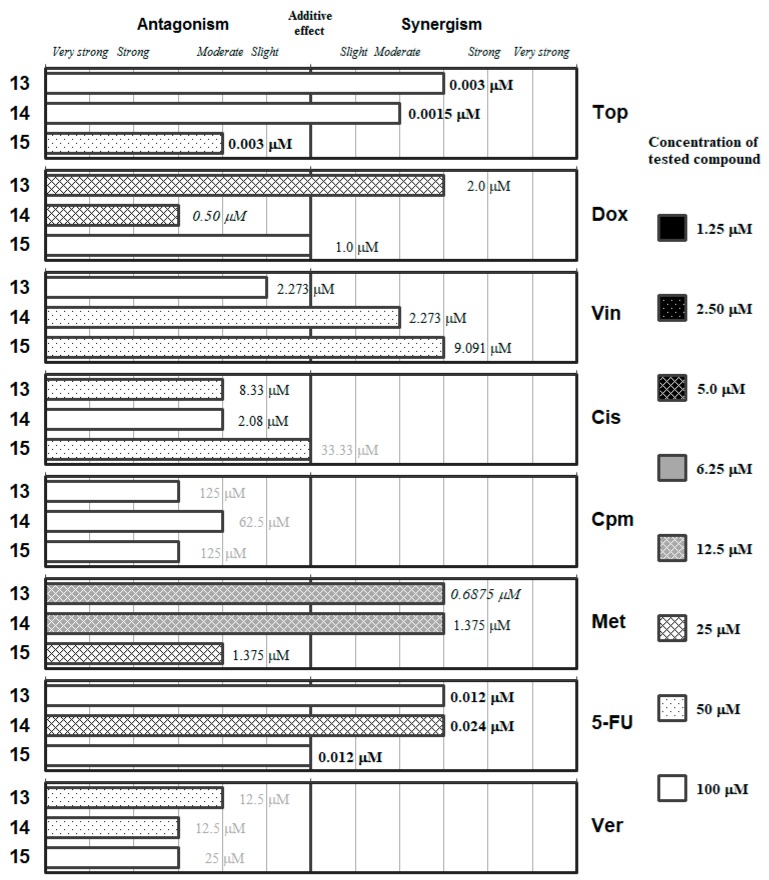
Interactions of the inorganic chalcogen cyanates (**13**–**15**) with a panel of anticancer drugs (*Top*, *Dox*, *Vin*, *Cis*, *Cpm*, *Met* and 5-*FU*) or an efflux pump inhibitor (*Ver*). Concentration of the tested compound is given according the legend; concentration of the anticancer drug is given in numbers inside the graph (in bold: below 0.1 µM, in italics between 0.1 µM and 1 µM, and in grey above 10 µM).

The detailed ratios and the concentrations of the most effective combinations are given in the Supplementary Material, in Tables S3–S10. These data are summarized in Figure 2, Figure 3, Figure 4, Figure 5 and Figure 6, which group the selenocompounds and the additional compounds/salts in relation to their chemical structure to ease the interpretation of their interactions with the panel of anticancer drugs. 

Figure 2 includes the phthalic selenoanhydride **1** and its oxygen isostere the phthalic anhydride (**12**). This Se-compound interacts synergistically with doxorubicin and with vincristine, being the optimal interaction at low concentrations of **1** (12.5 and 5 µM, respectively). These results suggest that **1** may interact with the microtubules for its synergy with *Vin*. Regarding *Dox*, its effect could be mediated by the generation of reactive oxygen species (ROS), as the selenocompounds can act either as antioxidants or as prooxidants. This additional mechanism of action of *Dox* seems more probable than the inhibition of the topoisomerase II enzyme, as the interaction of **1** with *Top* (a topoisomerase-I inhibitor) is slightly antagonistic. On the other hand, the phthalic anhydride **12** generally interacts with the different drugs (except for *Met* and *Cis*) at higher concentrations than its selenium isostere (**1**). Compound **12** only showed synergistic interactions with topotecan, vincristine and *5-FU*, but at high concentrations of **12** (25, 50 and 100 µM, respectively); its unique interaction at low concentration was with methotrexate (12.5 µM) and was moderately antagonistic. Interestingly, compound **12** showed additive effect with *Dox*, which supports the hypothesis of the ability of **1** to generate ROS thanks to the antioxidant/pro-oxidant properties of the selenium atom. On the other hand, verapamil and the selenoanhydride **1** interacted in an antagonistic manner, which is surprising taking into account that **1** was reported to be an efflux pump inhibitor (EPI) [42,43,44]. This may indicate a possible competition in the binding of the two EPIs (**1** and *Ver*) to the ABCB1 protein.

Symmetric bi-functionalized dimethyl selenodiesters **2**–**5** showed (with certain exceptions) synergistic or moderately synergistic interactions with verapamil and with most of the anticancer drugs evaluated, except for cisplatin, as shown in Figure 3. The drugs that showed more synergistic interaction (and at lower concentrations of the Se-compounds) were *Vin* and *Cpm*; whereas *Cis* interacted in an antagonistic manner and *5-FU* resulted in be the less synergistic of the remaining drugs, as it requires higher concentrations of the selenium derivatives. Compounds **2** and **4** showed a strong synergistic interaction with *Vin*, and at relatively low concentrations of the Se-compounds (12.5 and 6.25 µM, respectively), which can suggest that these Se-compounds can affect microtubule stability. 

It is noteworthy to highlight the contrast between the antagonistic interaction of **2**–**5** with *Cis* and the synergistic interaction of **2**–**5** with *Cpm*, indicating that the direct alkylation is more positive than the alkylating-like one of *Cis*. The symmetric thiophene derivative (**2**) may be the most adequate of the Se-compounds because it did not present with any antagonistic interactions and secondly it interacts in different synergistic degrees with *Top*, *Vin*, *Cpm,* and *Met* at a concentration of 12.5 µM. The dimethyl pyridine-1,6-dicarboselenoate **3** had a differential effect on the anticancer drugs: at concentrations in the range 6.25–12.5 µM, it showed differential antagonistic interactions with *Top*, *Cis* and *5-FU*, whereas it interacted in different degrees of synergism with *Dox*, *Vin,* and *Cpm*. Finally, between the benzene dicarboselenoates **4** and **5**, the *meta*-substituted (**4**) showed interactions at equal or lower concentration than the *para*-substituted (**5**), except in the case of *Top*. This suggests a possible influence of the substitution in their capacity to interact with anticancer drugs.

The results of the combination assay with anticancer drugs for the selenoesters containing amides (**6**) or oxygen esters (**7**,**8**) in their lateral chain indicated that none of these compounds exerted synergistic interactions with the anticancer drugs at concentrations of the Se-compound below 20 µM, thus limiting their potential application as chemotherapy adjuvants (Figure 4). 

The derivatives containing an oxygen ester (**7** and **8**) showed different grades of synergism with vincristine at a concentration of 25 µM, and **7** also showed moderate synergism with cyclophosphamide at this low concentration of Se-compound. The amide-containing derivative **6** only showed at this concentration (25 µM of Se-compound) a slight synergism with *Dox*. 

Nevertheless, out of the 15 compounds evaluated the ketone-containing Se-compounds **9**–**11** (Figure 5) interacted with the different anticancer drugs at the lowest concentrations: all of them showed interactions at Se-compounds concentrations in the range from 1.25 to 2.5 µM with the seven selected anticancer drugs. In the case of the verapamil, antagonistic interactions were observed at a higher concentration range for the Se-compounds (5–12.5 µM). 

The methyl-ketone derivative **9** exerted synergistic interactions with topotecan and vincristine at a concentration of Se-compound as low as 1.25 µM, as well as moderately synergistic enhancements of the activity of doxorubicin and cyclophosphamide at a concentration of 2.50 µM. In contrast, Se-compound **9** interacted in different grades of antagonism at concentration of 1.25 µM with *Cis*, *Met*, *5-FU*, and *Ver*. This differential behavior suggests that this ketone Se-compound may affect the topoisomerase enzymes (as both *Top* and *Dox* are topoisomerase inhibitors), inhibit the microtubule formation and mediate the direct alkylation of DNA (*Cpm*). Alternatively, this compound hinders the remaining mechanisms tested: the alkylation-like action of *Cis*, and the antimetabolite activity of *Met* and *5-FU* in the synthesis of folic acid and nucleotides, respectively. The antagonistic interaction between *Ver* and **9** is surprising, taking into account that this compound inhibited the ABCB1 efflux pump with a potency up to 4-fold of the inhibitory activity determined for *Ver* in previous studies [42,43,44]. This observation may suggest competition between the two ABCB1 inhibitors at the time of interacting with this transmembrane efflux pump. 

The two evaluated *tert*-butyl ketone selenoesters (**10** and **11**) showed a similar pattern of interaction with the anticancer drugs and with *Ver* as the methyl-ketone **9** described above, with some differences. Compound **10** interacted in different grades of synergism with *Top* and with *Vin* at a concentration of Se-compound of 1.25 µM, and with *Dox* at 2.5 µM. Compound **10** showed additive effect with *Cis*, and different grades of antagonism with *Cpm*, *Met*, *5-FU*, and *Ver*. On the other hand, compound **11** at a 2.5 µM concentration showed synergistic interactions with *Top* and *Vin*, additive effect with *Dox* and different grades of antagonism with the remaining drugs. The result of the interaction with verapamil is again surprising, as these two derivatives (**10** and **11**) were also potent inhibitors of the ABCB1 efflux pump in the previous experiments. Summing up, based on the results obtained, the *tert*-butyl ketone selenoesters have a more favorable interaction with the topoisomerase inhibitors *Top* and *Dox* and with the microtubule inhibitor *Vin*. 

The graphs representing the interactions between the inorganic XCN (X=O, S and Se for **13**, **14** and **15**, respectively) salts are provided in Figure 6. Overall, the oxygen salt and the sulfur salt seemed to have better interaction profile against the different drugs than the potassium selenocyanate (**15**), as the latter only improved the interaction of its oxygen/sulfur analogues in its interaction with *Vin* and with *Cis*, but in both cases at high concentration of the salts (50 µM). Only KOCN (**13**) and NH_4_SCN (**14**) were able to interact in a synergistic manner with an anticancer drug (*Met*) at a relevant concentration in the biological assays (12.5 µM). Besides that, **13** showed synergistic interaction with *Dox* and **14** with *5-FU* at a concentration of salt of 25 µM. On the other hand, the sulfur salt (**14**) showed an antagonistic effect with *Dox*, and the selenium salt a moderately antagonistic interaction with methotrexate at this 25 µM concentration of the respective salt. These data suggest that the oxygen and the sulfur salt could be potential antimetabolites in the synthesis of folic acid. 

Summing up from the perspective of the anticancer drugs used, different grades of synergistic interactions were mostly observed in the case of *Vin* (all selenocompounds, apart from derivative **6**), *Dox* (7 Se-compounds, all except **4**, **7**, **8** and **11**), while six compounds showed different grades of synergistic interactions with cyclophosphamide (**2**, **3**, **5** and **7**–**9**) and with methotrexate (**2**–**6** and **8**); and five with *Top* (**2**, **4** and **9**–**11**) and *5-FU* (**2** and **4**–**7**), respectively. These compounds exhibited their beneficial effects in the concentration range between 1.25–100 µM. In contrast, mostly antagonistic interactions were observed in the case of *Cis* (7 Se-compounds: **1**, **3**, **4**, **7**–**9** and **11**) and *Ver* (7 Se-compounds: **1**, **5**, **6** and **8**–**11**). As mentioned before, it is surprising that compounds with a known ability to inhibit efflux pumps showed different grades of antagonistic interactions with a known EPI as *Ver*. This could be explained considering that the two EPIs (*Ver* and the respective selenocompound) may have a competitive binding to the ABCB1 protein when ABCB1-overexpressing cell lines were treated with both compounds simultaneously. The tiophene-derivative selenoester (**2**) presented moderate-strong synergism (with CI values ranging between 0.20–0.78) in all tested drugs except with *Cis* (CI: 0.96), which suggests that the presence of the sulfur-containing heterocyclic hydrocarbon moiety in the structure of the tested compounds has a pivotal influence on the efficacy. Interestingly, compound **2** did not exhibit potent cytotoxic or efflux pump modulatory properties in our previous study, which suggests that their efficacy is associated with other mechanisms [42]. These results support our previous findings, in relation to organosulfur chalcogens from earlier studies, as those compounds presented with no pronounced anticancer or EPI modulatory activity, while displaying strong synergistic interactions with most of the tested drugs (unpublished results). Interestingly, there were cases, when the reference chalcogen compounds presented synergism with the anticancer drugs (*Dox*: **13**; *Top*: **12**–**14**; *Vin*: **14**–**15**; *5-FU*: **12**–**14**) in the 25–100 µM concentration range. However, the cyclic selenoanhydride (**1**) and phthalic anhydride (**12**) generally showed similar interaction profiles, irrespective of the nature of the chalcogen atom included in the molecule (O vs. Se), as relevant differences were only observed in the case of *Top* (slight antagonism vs. synergism).

These observed activities open a new promising approach to fight multidrug resistance in cancer. Nevertheless, we need to consider that this is a limited preliminary experiment that would require further studies in more complex models once that the safety of the compounds is established in parallel studies currently ongoing. While the underlying mechanisms are not yet elucidated, the potency of the tested selenocompounds as adjuvants may be attributable to their activities are effective modulators of apoptosis and ATP-dependent efflux pumps [42,49]. These pumps, owing to their wide substrate specificity, can extrude a variety of chemotherapeutic drugs, thus preventing them from reaching their cellular targets at effective concentrations [21,50,51,52]. Considering this, it is not surprising that favorable interactions were observed in relation to *Vin*, *Dox* and *Top*, all being major substrates of these multidrug efflux pumps (specifically ABCB1 in our model system) [53,54]. However, compounds **1** and the methyl-ketone selenoesters **9**–**11** (the most potent EPI inhibitors in previous studies) did not show superior efficacy in the combination assays (with CI values ranging between 0.41–2.81), thus it would be safe to assume that other mechanisms should play a role in their pharmacological interactions [43]. This is highlighted by the fact that interactions between the selenocompounds and verapamil (which exerts cytotoxic as well as pump inhibitor activity in the tested concentration) were negative for **13** out of **15** compounds. Nonetheless, it has been described that some chemotherapeutic drugs (with the most extensive literature on anthracyclines, vinca alkaloids and camptothecin-analogues in this respect) exert their antitumor activities not only by binding to specific molecular targets (i.e., topoisomerase-I/II enzyme, tubulin-microtubule system), but through a non-specific modulation of ROS, affecting various cellular components [34,55,56,57]. Therefore, it is feasible that the cumulative activity of these ROS-modulating effects might play a role in the interaction profile of the Se-compounds with the chemotherapeutics, potentiating their anticancer activity against drug resistant lymphoma cells [58].

## 3. Materials and Methods

### 3.1. Chemistry

The synthesis and characterization of the cyclic selenoanhydride (**1**) and the ten selenoesters investigated in our study (**2**–**11**) was previously described elsewhere [41]. To get the amount of compound needed for assays, the derivatives were re-synthesized. The 11 compounds were pure and chemically stable on air, according to the spectroscopic (IR, ^1^H- and ^13^C-NMR, MS) and the elemental analysis performed to confirm the structures of the different derivatives as reported in [41]. ^1^H and ^13^C-NMR of representative compounds shown in Supplementary Material (Figures S2–S9). The four chalcogen compounds (**12**–**15**; **12**-phthalic anhydride; the oxygen isoster of compound **1**, **13**-potassium cyanate, **14**-ammonium thiocyanate, **15**-potassium selenocyanate) used as references were purchased (Sigma-Aldrich, St Louis, MO, USA) [43]. Compounds were solved in DMSO to obtain stock solutions. Afterwards, working solutions were prepared by dilution in water, with the concentration of DMSO below 1% in all the experiments.

Other chemicals used in the study as reagents were: doxorubicin-hydrochloride (Wako Pure Chem. Ind., Osaka, Japan), cisplatin (TEVA, Petah Tikva, Israel), 5-fluorouracil (Accord, North Harrow, UK) topotecan (GlaxoSmithKline, Brentford, London), vincristine (Richter, Budapest, Hungary), cyclophosphamide (Baxter, Deerfield, IL, USA) verapamil (EGIS, Budapest, Hungary), methotrexate (Ebewe Pharmaceutical Company, Unterach, Austria), 3-(4,5-dimethylthiazol-2-yl)-2,5-diphenyltetrazolium bromide (MTT; Sigma-Aldrich, St Louis, MO, USA), sodium dodecyl sulfate (SDS; Sigma) and dimethyl-sulfoxide (DMSO; Sigma-Aldrich, St Louis, MO, USA). All solutions were prepared on the day of assay.

### 3.2. Cell Lines

L5178Y mouse T-cell lymphoma cells (PAR) (ECACC Cat. No. 87111908, obtained from FDA, Silver Spring, MD, USA) were transfected with pHa MDR1/A retrovirus, as previously described by Cornwell et al. [59]. The ABCB1-expressing cell line L5178Y (MDR) was selected by culturing the infected cells with colchicine. The cells were cultured in McCoy’s 5A medium (Sigma-Aldrich, St Louis, MO, USA) supplemented with 10% heat-inactivated horse serum (Sigma-Aldrich, St Louis, MO, USA), 200 mM L glutamine (Sigma-Aldrich, St Louis, MO, USA), nystatin and a penicillin-streptomycin (Sigma-Aldrich, St Louis, MO, USA) mixture in concentrations of 100 IU/L and 10 mg/L, respectively.

### 3.3. Checkerboard Combination Assay

A checkerboard microplate method was applied to study the effect of drug interactions between the selenocompounds (**1**–**11**), chalcogen compounds (**12**–**15**) and the reference chemotherapeutic drugs as well as verapamil [60]. The agents were chosen to include several compounds with diverse mechanisms of action, while verapamil was included because this compound was used as a positive control in our previous experiments regarding the efflux pump inhibitory properties of these compounds [42,43,44]. The assay was carried out using multidrug-resistant (MDR) mouse t-lymphoma cells overexpressing the ABCB1 transporter. The final concentration of the chemotherapeutic agents used in the combination experiment was chosen in accordance with their cytotoxicity on parental and multidrug-resistant mouse t-lymphoma cells, while the final concentrations of the selenocompounds used were based on our previous study (for the concentrations of the stock solutions used and the final concentrations, see the Supplementary material) [42]. The dilutions of the chemotherapeutic drugs (or verapamil) were made in a horizontal direction in 100 μL, and the dilutions of the selenocompounds vertically in the microtiter plate in 50 μL volume (see Figure S1. in Supplementary material). The cells were re-suspended in culture medium and distributed into each well in 50 μL containing 6 × 10^3^ cells each. The plates were incubated for 72 h at 37 °C in 5% CO_2_ atmosphere. The cell growth rate was determined after MTT staining. At the end of the incubation period, 20 μL of MTT (thiazolyl blue tetrazolium bromide, Sigma) solution (from a stock solution of 5 mg/mL) were added to each well. After incubation at 37 °C for 4 h, 100 μL of SDS (Sigma-Aldrich, St. Louis, MO, USA) solution (10% in 0.01 M HCl; Merck, Darmstadt, Germany) were added to each well and the plates were further incubated at 37 °C overnight. Optical density (OD) was measured at 540/630 nm with Multiscan EX ELISA reader (Thermo Labsystems, Cheshire, WA, USA) as described elsewhere [60]. Combination index (CI) values at 50% of the growth inhibition dose (ED_50_), were determined using CompuSyn software (ComboSyn, Inc., Paramus, NJ, USA) to plot four to five data points to each ratio [47,61]. CI values were calculated by means of the median-effect equation, according to the Chou-Talalay method, where CI < 1, CI = 1, and CI > 1 represent synergism, additive effect (or no interaction), and antagonism, respectively (see Supplementary material) [47,48]. Results are graphically shown in Figure 2, Figure 3, Figure 4, Figure 5 and Figure 6 in the previous section (2. Results and Discussion). Detailed results, with the corresponding standard deviations, are given in Supplementary Material (Tables S3–S10).

## 4. Conclusions

Herein we have evaluated the capacity of a series of selenocompounds to interact with seven different chemotherapy drugs (topotecan, doxorubicin, vincristine, cisplatin, cyclophosphamide, methotrexate and 5-fluorouracil) and the efflux pump inhibitor verapamil, using a checkerboard microplate method and a MDR mouse t-lymphoma cell line. The results indicated that the selenocompounds showed a marked capacity to interact with the anticancer drugs tested. Vincristine was the chemotherapy drug that showed different grades of synergistic interactions with the highest number of selenocompounds (10), followed by doxorubicin (7 Se-compounds), cyclophosphamide and methotrexate (6 Se-compounds), topotecan and 5-fluorouracil (5 Se-compounds); whereas interactions with cisplatin and verapamil were mostly antagonistic. These observations suggest that the tested Se-compounds can interact with the formation of microtubules and with the action of the cellular topoisomerase enzymes. 

On the other hand, the interaction of Se-compounds that were proven to be active as efflux pump inhibitors in previous studies was antagonistic with the efflux pump inhibitor verapamil, which suggests a possible competition among the two classes of efflux inhibitors at the time of their interaction with their target, the ABCB1 protein. Regarding how the functional groups present in the Se-compounds affect to the interaction, the symmetrical compounds **2**–**5**, which contain two selenium atoms, interacted in a synergistic manner with the highest number of chemotherapy drugs, the thiophene-containing Se-compound **2** was the most efficient in this regard, as it showed synergistic interactions with all tested drugs except cisplatin. In terms of concentration, the selenoesters **9**–**11** (those which contain a ketone in the alkyl chain bound to the selenoester) were able to interact with topotecan (**9**–**11**), vincristine (**9**–**11**), doxorubicin (**9** and **10**) and cyclophosphamide (**9**) at concentrations as low as 1.25 µM or 2.5 µM.

Based on our results, these selenocompounds are a promising new class of potential adjuvants of chemotherapy drugs, that can be used as a novel approach to fight the increasing and troublesome multidrug resistance in cancer. Although this is an initial work and further research needs to be carried out for the more-in-depth exploration of the potential applications of these compounds and of their derivatives that enable the optimization of their desired activities. 

## Figures and Tables

**Table 1:** Summary of interaction types related to combination index (CI) values [48].

Combination Index (CI)	Type of Interaction	Combination Index (CI)	Type of Interaction
0–0.1	very strong synergism	***0.9*** *–**1.1***	***additive effect***
0.1–0.3	strong synergism	1.1–1.2	slight antagonism
***0.3***–***0.7***	***synergism***	1.2–1.45	moderate antagonism
		***1.45***–***3.3***	***antagonism***
0.7–0.85	moderate synergism	3.3–10	strong antagonism
0.85–0.9	slight synergism	>10	very strong antagonism

## Supplementary Materials

The following materials are available online, Tables S1 and S2: Chemotherapeutic agents and selenocompounds tested; Tables S3–S10: Interactions between selenocompounds and each different chemotherapeutic drug evaluated; Figure S1: Arrangement of 96-well microtiter plates for checkerboard combination assay; Figures S2–S5: ^1^H-NMR spectra of selected compounds; Figures S6–S9: ^13^C-NMR spectra of selected compounds.

## Abbreviations:

*5-FU*	5-fluorouracil
ABCB1	ATP-binding cassette transporter subfamily B member 1
CI	combination index
*Cis*	cisplatin
*Cpm*	cyclophosphamide
DMSO	dimethyl-sulfoxide
*Dox*	doxorubicin
EPI	efflux pump inhibitor
EU	European Union
FDA	Food and Drug Administration
IU	international units
IR	infrared spectroscopy
MDR	multidrug-resistant/multidrug resistance
MS	mass spectrometry
MTT	3-(4,5-dimethylthiazol-2-yl)-2,5-diphenyltetrazolium bromide
*Mtx*	methotrexate
NMR	nuclear magnetic resonance
OD	optical density
PAR	parental cell line
SDS	sodium dodecyl sulfate
QoL	Quality of Life
US	United States
*Ver*	verapamil
*Vin*	vincristine

## References

[1] McGuire S. (2016). World Cancer Report 2014. Geneva, Switzerland: World Health Organization, International Agency for Research on Cancer, WHO Press, 2015. Adv. Nutr..

[2] Munro A.J. (2014). Comparative cancer survival in European countries. Br. Med. Bull..

[3] Hermine O., Ramos J.C., Tobinai K. (2018). A Review of New Findings in Adult T-cell Leukemia-Lymphoma: A Focus on Current and Emerging Treatment Strategies. Adv. Ther..

[4] Suzuki R. (2018). NK/T Cell Lymphoma: Updates in Therapy. Curr. Hematol. Malig. Rep..

[5] Siaghani P.J., Song J.Y. (2018). Updates of Peripheral T Cell Lymphomas Based on the 2017 WHO Classification. Curr. Hematol. Malig. Rep..

[6] Leonard J., Stock W. (2017). Progress in adult ALL: Incorporation of new agents to frontline treatment. Hematol. Am. Soc. Hematol. Educ. Program.

[7] Lue J.K., Kress A., Amengual J.E. (2017). Therapeutic Options for Aggressive T-Cell Lymphomas. Curr. Hematol. Malig. Rep..

[8] Subbiah V., Kurzrock R. (2018). Challenging Standard-of-Care Paradigms in the Precision Oncology Era. Trends Cancer.

[9] Sumpio C., Knobf M.T., Jeon S. (2016). Treatment complexity: A description of chemotherapy and supportive care treatment visits in patients with advanced-stage cancer diagnoses. Support. Care Cancer.

[10] Chabner B.A., Roberts T.G. (2005). Timeline: Chemotherapy and the war on cancer. Nat. Rev. Cancer.

[11] Govender J., Loos B., Marais E., Engelbrecht A.-M. (2014). Mitochondrial catastrophe during doxorubicin-induced cardiotoxicity: A review of the protective role of melatonin. J. Pineal Res..

[12] Ichikawa Y., Ghanefar M., Bayeva M., Wu R., Khechaduri A., Naga Prasad S.V., Mutharasan R.K., Naik T.J., Ardehali H. (2014). Cardiotoxicity of doxorubicin is mediated through mitochondrial iron accumulation. J. Clin. Investig..

[13] Octavia Y., Tocchetti C.G., Gabrielson K.L., Janssens S., Crijns H.J., Moens A.L. (2012). Doxorubicin-induced cardiomyopathy: From molecular mechanisms to therapeutic strategies. J. Mol. Cell. Cardiol..

[14] Robinson D., Schulz G., Langley R., Donze K., Winchester K., Rodgers C. (2014). Evidence-Based Practice Recommendations for Hydration in Children and Adolescents with Cancer Receiving Intravenous Cyclophosphamide. J. Pediatr. Oncol. Nurs..

[15] Au W., Sokova O.I., Kopnin B., Arrighi F.E. (1980). Cytogenetic toxicity of cyclophosphamide and its metabolites in vitro. Cytogenet. Cell Genet..

[16] Acton E.M., Narayanan V.L., Risbood P.A., Shoemaker R.H., Vistica D.T., Boyd M.R. (1994). Anticancer specificity of some ellipticinium salts against human brain tumors in vitro. J. Med. Chem..

[17] Bayat Mokhtari R., Homayouni T.S., Baluch N., Morgatskaya E., Kumar S., Das B., Yeger H. (2017). Combination therapy in combating cancer. Oncotarget.

[18] Piccolo M.T., Menale C., Crispi S. (2015). Combined anticancer therapies: An overview of the latest applications. Anticancer Agents Med. Chem..

[19] Baguley B.C. (2010). Multidrug resistance in cancer. Methods Mol. Biol..

[20] Yardley D.A. (2013). Drug Resistance and the Role of Combination Chemotherapy in Improving Patient Outcomes. Int. J. Breast Cancer.

[21] Moitra K., Lou H., Dean M. (2011). Multidrug efflux pumps and cancer stem cells: Insights into multidrug resistance and therapeutic development. Clin. Pharmacol. Ther..

[22] Li Y.-J., Lei Y.-H., Yao N., Wang C.-R., Hu N., Ye W.-C., Zhang D.-M., Chen Z.-S. (2017). Autophagy and multidrug resistance in cancer. Chin. J. Cancer.

[23] Teicher B.A., Herman T.S., Holden S.A., Wang Y.Y., Pfeffer M.R., Crawford J.W., Frei E. (1990). Tumor resistance to alkylating agents conferred by mechanisms operative only in vivo. Science.

[24] Mecklenburg S., Shaaban S., Ba L.A., Burkholz T., Schneider T., Diesel B., Kiemer A.K., Röseler A., Becker K., Reichrath J. (2009). Exploring synthetic avenues for the effective synthesis of selenium- and tellurium-containing multifunctional redox agents. Org. Biomol. Chem..

[25] Jamier V., Ba L.A., Jacob C. (2010). Selenium- and tellurium-containing multifunctional redox agents as biochemical redox modulators with selective cytotoxicity. Chemistry.

[26] Sanmartin C., Plano D., Font M., Palop J.A. (2011). Selenium and clinical trials: New therapeutic evidence for multiple diseases. Curr. Med. Chem..

[27] Plano D., Baquedano Y., Ibáñez E., Jiménez I., Palop J.A., Spallholz J.E., Sanmartín C. (2010). Antioxidant-prooxidant properties of a new organoselenium compound library. Molecules.

[28] Bartolini D., Sancineto L., Fabro de Bem A., Tew K.D., Santi C., Radi R., Toquato P., Galli F. (2017). Selenocompounds in Cancer Therapy: An Overview. Adv. Cancer Res..

[29] Moreno E., Plano D., Lamberto I., Font M., Encío I., Palop J.A., Sanmartín C. (2012). Sulfur and selenium derivatives of quinazoline and pyrido[2,3-d]pyrimidine: Synthesis and study of their potential cytotoxic activity in vitro. Eur. J. Med. Chem..

[30] Plano D., Sanmartín C., Moreno E., Prior C., Calvo A., Palop J.A. (2007). Novel potent organoselenium compounds as cytotoxic agents in prostate cancer cells. Bioorg. Med. Chem. Lett..

[31] Álvarez-Pérez M., Ali W., Marć M.A., Handzlik J., Domínguez-Álvarez E. (2018). Selenides and Diselenides: A Review of Their Anticancer and Chemopreventive Activity. Molecules.

[32] Gandin V., Khalkar P., Braude J., Fernandes A.P. (2018). Organic selenium compounds as potential chemotherapeutic agents for improved cancer treatment. Free Radic. Biol. Med..

[33] Cort A., Ozben T., Saso L., De Luca C., Korkina L. (2016). Redox Control of Multidrug Resistance and Its Possible Modulation by Antioxidants. Oxid. Med. Cell. Longev..

[34] Peng X., Gandhi V. (2012). ROS-activated anticancer prodrugs: A new strategy for tumor-specific damage. Ther. Deliv..

[35] Park S.O., Yoo Y.B., Kim Y.H., Baek K.J., Yang J.-H., Choi P.C., Lee J.H., Lee K.R., Park K.S. (2015). Effects of combination therapy of docetaxel with selenium on the human breast cancer cell lines MDA-MB-231 and MCF-7. Ann. Surg. Treat. Res..

[36] Kumi-Diaka J., Merchant K., Haces A., Hormann V., Johnson M. (2010). Genistein-selenium combination induces growth arrest in prostate cancer cells. J. Med. Food.

[37] Chakraborty P., Roy S.S., Bhattacharya S. (2015). Molecular mechanism behind the synergistic activity of diphenylmethyl selenocyanate and Cisplatin against murine tumor model. Anticancer Agents Med. Chem..

[38] Chen T., Wong Y.-S. (2009). Selenocystine induces caspase-independent apoptosis in MCF-7 human breast carcinoma cells with involvement of p53 phosphorylation and reactive oxygen species generation. Int. J. Biochem. Cell Biol..

[39] Chakraborty P., Roy S.S., Basu A., Bhattacharya S. (2016). Sensitization of cancer cells to cyclophosphamide therapy by an organoselenium compound through ROS-mediated apoptosis. Biomed. Pharmacother..

[40] Qi Y., Fu X., Xiong Z., Zhang H., Hill S.M., Rowan B.G., Dong Y. (2012). Methylseleninic acid enhances paclitaxel efficacy for the treatment of triple-negative breast cancer. PLoS ONE.

[41] Domínguez-Álvarez E., Plano D., Font M., Calvo A., Prior C., Jacob C., Palop J.A., Sanmartín C. (2014). Synthesis and antiproliferative activity of novel selenoester derivatives. Eur. J. Med. Chem..

[42] Domínguez-Álvarez E., Gajdács M., Spengler G., Palop J.A., Marć M.A., Kieć-Kononowicz K., Amaral L., Molnár J., Jacob C., Handzlik J. (2016). Identification of selenocompounds with promising properties to reverse cancer multidrug resistance. Bioorg. Med. Chem. Lett..

[43] Gajdács M., Spengler G., Sanmartín C., Marć M.A., Handzlik J., Domínguez-Álvarez E. (2017). Selenoesters and selenoanhydrides as novel multidrug resistance reversing agents: A confirmation study in a colon cancer MDR cell line. Bioorg. Med. Chem. Lett..

[44] Gajdács M., Handzlik J., Sanmartin C., Domínguez E., Spengler G. (2018). [Organoselenium compounds as antitumor agents: In vitro evaluation on a colon cancer model system] (article in Hungarian). Acta Pharm. Hung..

[45] Gajdács M., Handzlik J., Sanmartín C., Domínguez-Álvarez E., Spengler G. (2018). [Prediction of ADME properties for selenocompounds with anticancer and efflux pump inhibitory activity using preliminary computational methods] (article in Hungarian). Acta Pharm. Hung..

[46] Chou T.-C. (2008). Preclinical versus clinical drug combination studies. Leuk. Lymphoma.

[47] Chou T.-C. (2006). Theoretical basis, experimental design, and computerized simulation of synergism and antagonism in drug combination studies. Pharmacol. Rev..

[48] Chou T.-C. (2010). Drug Combination Studies and Their Synergy Quantification Using the Chou-Talalay Method. Cancer Res..

[49] Sanmartín C., Plano D., Sharma A.K., Palop J.A. (2012). Selenium Compounds, Apoptosis and Other Types of Cell Death: An Overview for Cancer Therapy. Int. J. Mol. Sci..

[50] Kathawala R.J., Gupta P., Ashby C.R., Chen Z.-S. (2015). The modulation of ABC transporter-mediated multidrug resistance in cancer: A review of the past decade. Drug Resist. Updat..

[51] Fletcher J.I., Haber M., Henderson M.J., Norris M.D. (2010). ABC transporters in cancer: More than just drug efflux pumps. Nat. Rev. Cancer.

[52] Spengler G., Evaristo M., Handzlik J., Serly J., Molnár J., Viveiros M., Kiéc-Kononowicz K., Amaral L. (2010). Biological activity of hydantoin derivatives on P-glycoprotein (ABCB1) of mouse lymphoma cells. Anticancer Res..

[53] Joyce H., McCann A., Clynes M., Larkin A. (2015). Influence of multidrug resistance and drug transport proteins on chemotherapy drug metabolism. Expert Opin. Drug Metab. Toxicol..

[54] Kobori T., Harada S., Nakamoto K., Tokuyama S. (2014). Mechanisms of P-glycoprotein alteration during anticancer treatment: Role in the pharmacokinetic and pharmacological effects of various substrate drugs. J. Pharmacol. Sci..

[55] Molavian H.R., Goldman A., Phipps C.J., Kohandel M., Wouters B.G., Sengupta S., Sivaloganathan S. (2016). Drug-induced reactive oxygen species (ROS) rely on cell membrane properties to exert anticancer effects. Sci. Rep..

[56] Yokoyama C., Sueyoshi Y., Ema M., Mori Y., Takaishi K., Hisatomi H. (2017). Induction of oxidative stress by anticancer drugs in the presence and absence of cells. Oncol. Lett..

[57] Briehl M.M., Tome M.E., Wilkinson S.T., Jaramillo M.C., Lee K. (2014). Mitochondria and redox homeostasis as chemotherapeutic targets. Biochem. Soc. Trans..

[58] Graczyk-Jarzynka A., Zagozdzon R., Muchowicz A., Siernicka M., Juszczynski P., Firczuk M. (2017). New insights into redox homeostasis as a therapeutic target in B-cell malignancies. Curr. Opin. Hematol..

[59] Cornwell M.M., Pastan I., Gottesman M.M. (1987). Certain calcium channel blockers bind specifically to multidrug-resistant human KB carcinoma membrane vesicles and inhibit drug binding to P-glycoprotein. J. Biol. Chem..

[60] Takács D., Csonka Á., Horváth Á., Windt T., Gajdács M., Riedl Z., Hajós G., Amaral L., Molnár J., Spengler G. (2015). Reversal of ABCB1-related Multidrug Resistance of Colonic Adenocarcinoma Cells by Phenothiazines. Anticancer Res..

[61] Chou T., Martin N. (2005). CompuSyn for Drug Combinations: PC Software and User’s Guide: A Computer Program for Quantitation of Synergism and Antagonism in Drug Combinations, and the Determination of IC50 and ED50 and LD50 Values.

