# Supplementary material for: Organoselenium Compounds as Novel Adjuvants of Chemotherapy Drugs—A Promising Approach to Fight Cancer Drug Resistance
Source: Molecules. 2019 Jan 18;24(2):336. doi: 10.3390/molecules24020336 (PMC6359065; doi:10.3390/molecules24020336)
Supplement: Supplementary file 1 [file molecules-24-00336-s001.pdf]

Organoselenium compounds as novel adjuvants of chemotherapy drugs: a promising approach to fight cancer drug resistance.

Gabriella Spengler[1,†], Márió Gajdács[1,†], Małgorzata Anna Marć[2], Enrique Domínguez-Álvarez[3*], Carmen Sanmartín[4,5*]

1 Department of Medical Microbiology and Immunobiology, Faculty of Medicine, University of Szeged;
2 Interdisciplinary Excellence Centre, Department of Inorganic and Analytical Chemistry, University of Szeged
3 Instituto de Química Orgánica General, Consejo Superior de Investigaciones Científicas (IQOG-CSIC), Madrid, Spain
4 Department of Pharmaceutical Technology and Chemistry, School of Pharmacy and Nutrition, University of Navarra, Pamplona, Spain
5 Instituto de Investigaciones Sanitarias de Navarra (IdiSNA), Pamplona, Spain

Supplementary Material

Contents:

1. Chemotherapeutic agents and selenocompounds tested (Tables S1 and S2, respectively).

2. Interactions between selenocompounds and each different chemotherapeutic drug evaluated (Tables S3-S10)

3. Arrangement of 96-well microtiter plates for checkerboard combination assay (Figure S1).

4. ^{1}H-NMR spectra of selected compounds (Figures S2-S5).

5. ^{13}C-NMR spectra of selected compounds (Figures S6-S9).

Table S1.: Chemotherapeutic agents tested: stock solutions and final concentrations.

Reference drug	Mechanism of action	Concentration of stock solution	Final concentration in the combination assay
**Topotecan (*Topo*)**	*Topoisomerase-I-inhibitor*	1.0 mg/mL	0.024 μM
**Doxorubicin (*Dox*)**	*Topoisomerase-II-inhibitor, ROS species generation*	2.0 mg/mL	17.242 μM
**Vincristine (*Vin*)**	*Inhibition of microtubule formation*	1.0 mg/mL	36.363 μM
**Cisplatin (*Cis*)**	*Alkylating-like agent (interstrand and intrastrand cross-links with purine bases at 1,2-positions within DNA strands)*	0.5 mg/mL	33.333 μM
**Cyclophosphamide (*Cpm*)**	*Alkylating agent (interstrand and intrastrand cross-links at N-7 position within DNA strands)*	25 mg/mL	250.000 μM
**5-fluorouracil (*5-FU*)**	*Pyrimidine-antagonist*	50.0 mg/mL	0.192 μM
**Methotrexate (*Mtx*)**	*Purine-antagonist*	10.0 mg/mL	11.000 μM
**Verapamil (*Ver*)**	*Efflux pump inhibitor (Ca^{2+}-channel blocker)*	2.5 mg/mL	200.000 μM

Table S2.: Selenocompounds tested in combination assay: stock solutions and final concentrations.

Tested compounds	Concentration of stock solution	Final concentration in the combination assay
**1**	10 mM	25.0 μM
**2**		25.0 μM
**3**		50.0 μM
**4**		100.0 μM
**5**		100.0 μM
**6**		100.0 μM
**7**		100.0 μM
**8**		100.0 μM
**9**		10.0 μM
**10**		10.0 μM
**11**		10.0 μM
**12**		100.0 μM
**13**		100.0 μM
**14**		100.0 μM
**15**		100.0 μM

Table S3. Interactions between selenocompounds and topotecan (*Top*) against multidrug resistant mouse T-lymphoma cells

Compound	Ratio [µM] (*Topo* : Se)	CI	SD ±	Type of interaction
1	0.006 : 12.500	1.19	0.07	Slight antagonism
2	0.006 : 12.500	0.78	0.10	**Moderate synergism**
3	0.0015 : 12.5000	1.76	0.28	Antagonism
4	0.003 : 50.000	0.72	0.11	**Moderate synergism**
5	0.0015 : 25.0000	0.95	0.12	*Additive effect*
6	0.006 : 100.000	1.84	0.26	Antagonism
7	0.006 : 50.000	1.26	0.08	Moderate antagonism
8	0.0015 : 50.0000	1.24	0.10	Moderate antagonism
9	0.024 : 1.250	0.49	0.21	**Synergism**
10	0.0015 : 1.2500	0.51	0.13	**Synergism**
11	0.0015 : 2.5000	0.77	0.09	**Moderate synergism**
12	0.0015 : 25.000	0.41	0.06	**Synergism**
13	0.003 : 100.000	0.68	0.08	**Synergism**
14	0.0015 : 100.0000	0.74	0.06	**Moderate synergism**
15	0.003 : 50.000	1.22	0.06	Moderate antagonism

Table S4. Interactions between selenocompounds and doxorubicin (*Dox*) against multidrug resistant mouse T-lymphoma cells

Compound	Ratio [µM] (*Doxo* : Se)	CI	SD ±	Type of interaction
1	1 : 5	0.64	0.20	**Synergism**
2	1 : 50	0.42	0.08	**Synergism**
3	1 : 12.5	0.53	0.09	**Synergism**
4	8 : 50	1.03	0.09	*Additive effect*
5	2 : 100	0.61	0.09	**Synergism**
6	4 : 25	0.88	0.06	**Slight synergism**
7	2 : 50	1.48	0.31	Antagonism
8	8 : 50	1.15	0.02	Slight antagonism
9	1 : 2.5	0.81	0.14	**Moderate synergism**
10	1 : 2.5	0.83	0.07	**Moderate synergism**
11	2 : 2.5	1.04	0.13	*Additive effect*
12	1 : 100	1.04	0.04	*Additive effect*
13	2 : 25	0.47	0.22	**Synergism**
14	0.5 : 25	2.63	0.54	Antagonism
15	1 :100	0.97	0.08	*Additive effect*

Table S5. Interactions between selenocompounds and vincristine (Vin) against multidrug resistant mouse T-lymphoma cells

Compound	Ratio [µM] (*Vin* : Se)	CI	SD ±	Type of interaction
1	2.273 : 12.500	0.57	0.15	**Synergism**
2	9.091 : 12.500	0.20	0.01	**Strong synergism**
3	18.182 : 12.500	0.46	0.15	**Synergism**
4	9.091 : 6.250	0.27	0.07	**Strong synergism**
5	4.545 : 50.000	0.51	0.09	**Synergism**
6	2.273 : 100.000	1.26	0.14	Moderate antagonism
7	4.545 : 25.000	0.58	0.11	**Synergism**
8	4.545 : 25.000	0.80	0.06	**Moderate synergism**
9	1.136 : 1.250	0.39	0.01	**Synergism**
10	1.136 : 1.250	0.78	0.13	**Moderate synergism**
11	4.545 : 2.500	0.56	0.13	**Synergism**
12	2.273 : 100.000	0.68	0.10	**Synergism**
13	2.273 : 100.000	1.16	0.04	Slight antagonism
14	2.273 : 50.000	0.70	0.13	**Moderate synergism**
15	9.091 : 50.000	0.36	0.08	**Synergism**

Table S6. Interactions between selenocompounds and cisplatin (*Cis*) against multidrug resistant mouse T-lymphoma cells

Compound	Ratio [µM] (*Cis* : Se)	CI	SD ±	Type of interaction
1	4.16 : 25.00	1.26	0.11	Moderate antagonism
2	4.16 : 100.00	0.96	0.04	*Additive effect*
3	1.04 : 6.25	1.31	0.07	Moderate antagonism
4	1.04 : 12.50	4.09	0.25	Strong antagonism
5	4.16 : 100.00	1.01	0.08	*Additive effect*
6	8.33 : 100.00	0.99	0.10	*Additive effect*
7	33.33 : 25.00	1.56	0.19	Antagonism
8	8.33 : 100.00	2.25	0.16	Antagonism
9	4.16 : 1.25	2.17	0.28	Antagonism
10	2.08 : 2.50	1.03	0.03	*Additive effect*
11	8.33 : 1.25	1.21	0.17	Slight antagonism
12	8.33 : 25.00	1.42	0.17	Moderate antagonism
13	8.33 : 50.00	1.28	0.22	Moderate antagonism
14	2.08 : 100.00	1.39	0.14	Moderate antagonism
15	33.33 : 50.00	1.10	0.08	*Additive effect*

Table S7. Interactions between selenocompounds and cyclophosphamide (*Cpm*) against multidrug resistant mouse T-lymphoma cells

Compound	Ratio [µM] (*Cpm* : Se)	CI	SD ±	Type of interaction
1	125 : 6.25	1.40	0.16	Moderate antagonism
2	125 : 12.5	0.67	0.17	**Synergism**
3	125 : 12.5	0.61	0.16	**Synergism**
4	125 : 12.5	1.08	0.06	*Additive effect*
5	62.5 : 50	0.64	0.11	**Synergism**
6	62.5 : 50	1.39	0.16	Moderate antagonism
7	125 : 25	0.71	0.11	Moderate synergism
8	125 : 100	0.63	0.02	**Synergism**
9	125 : 2.5	0.75	0.10	Moderate synergism
10	125 : 2.5	1.43	0.14	Moderate antagonism
11	125 : 1.25	1.31	0.11	Moderate antagonism
12	125 : 25	1.55	0.08	Antagonism
13	125 : 100	1.58	0.39	Antagonism
14	62.5 : 100	1.39	0.25	Moderate antagonism
15	125 : 100	1.85	0.20	Antagonism

Table S8. Interactions between selenocompounds and methotrexate (*Met*) against multidrug resistant (MDR) mouse T-lymphoma cells

Compound	Ratio [µM] (*Met* : Se)	CI	SD ±	Type of interaction
1	0.6875:50	2.03	0.46	Antagonism
2	11:12.5	0.36	0.05	**Synergism**
3	2.75:25	0.71	0.02	**Moderate synergism**
4	1.375:25	0.71	0.16	**Moderate synergism**
5	0.6875:100	0.48	0.09	**Synergism**
6	5.5:100	1.40	0.16	**Slight synergism**
7	1.375:100	1.36	0.21	Moderate antagonism
8	1.375:100	0.68	0.16	**Synergism**
9	1.375:1.25	3.24	0.42	Antagonism
10	11:2.5	2.41	0.33	Antagonism
11	0.3438:1.25	2.26	0.39	Antagonism
12	5.5:12.5	1.34	0.13	Moderate antagonism
13	0.6875:12.5	0.59	0.05	**Synergism**
14	1.375:12.5	0.39	0.14	**Synergism**
15	1.375:25	1.33	0.09	Moderate antagonism

Table S9. Interactions between selenocompounds and 5-fluorouracil (*5-FU*) against multidrug resistant mouse T-lymphoma cells

Compound	Ratio [μM] (*5-FU* : Se)	CI	SD ±	Type of interaction
1	0.012 : 6.250	0.97	0.05	*Additive effect*
2	0.024 : 100.000	0.77	0.03	**Moderate synergism**
3	0.024 : 25.000	1.48	0.22	Antagonism
4	0.096 : 50.000	0.48	0.05	**Synergism**
5	0.024 : 100.000	0.42	0.14	**Synergism**
6	0.012 : 100.000	0.68	0.20	**Synergism**
7	0.012 : 100.000	0.83	0.07	**Moderate synergism**
8	0.024 : 50.000	1.34	0.11	Moderate antagonism
9	0.096 : 1.250	2.81	0.29	Antagonism
10	0.048 : 1.250	2.54	0.48	Antagonism
11	0.096 : 1.250	2.01	0.41	Antagonism
12	0.012 : 100.000	0.46	0.18	**Synergism**
13	0.012 : 100.000	0.42	0.07	**Synergism**
14	0.024 : 25.000	0.69	0.14	**Synergism**
15	0.012 : 100.000	1.06	0.05	*Additive effect*

Table S10. Interactions between selenocompounds and verapamil (*Ver*) against multidrug resistant mouse T-lymphoma cells

Compound	Ratio [μM] (*Ver* : Se)	CI	SD ±	Type of interaction
1	12.5 : 12.5	1.67	0.28	Antagonism
2	25 : 25	0.67	0.09	**Synergism**
3	12.5 : 25	0.57	0.19	**Synergism**
4	25 : 25	1.05	0.03	*Additive effect*
5	6.25 : 25	1.98	0.38	Antagonism
6	50 : 100	1.89	0.26	Antagonism
7	12.5 : 50.0	1.01	0.07	*Additive effect*
8	6.25 : 12.50	2.30	0.54	Antagonism
9	25.0 : 12.5	1.41	0.13	Moderate antagonism
10	50 : 5	1.56	0.29	Antagonism
11	25 : 12.5	1.73	0.30	Antagonism
12	50 : 50	1.18	0.04	Slight antagonism
13	12.5 : 50	1.43	0.12	Moderate antagonism
14	12.5 : 50	1.91	0.36	Antagonism
15	25 : 100	1.68	0.14	Antagonism

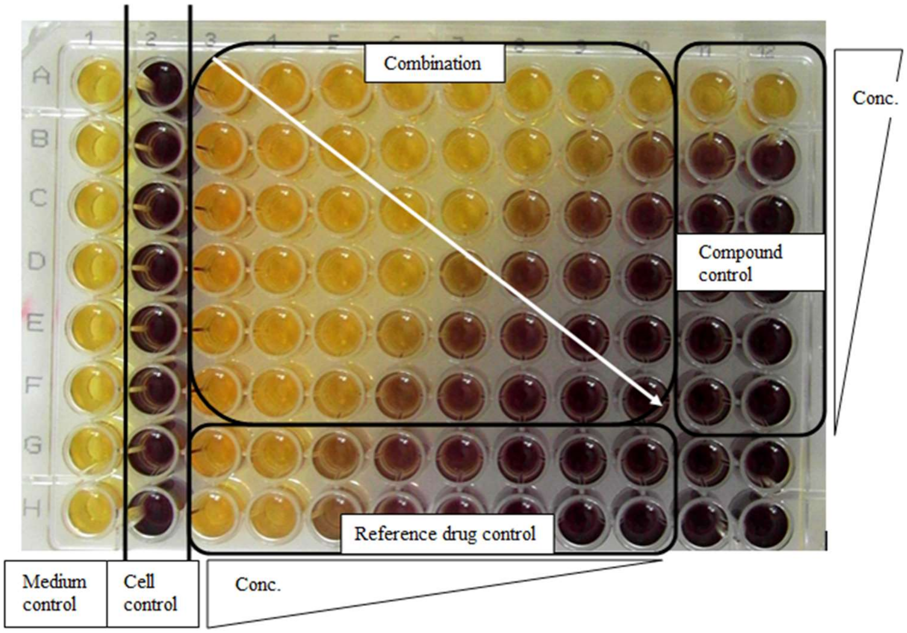

Figure 1. Arrangement of 96-well microtiter plates for checkerboard combination assay. The dilutions of the chemotherapeutic drugs (or verapamil) were made in a horizontal direction in 100 μL, and the dilutions of the selenocompounds vertically in the microtiter plate in 50 μL volume.

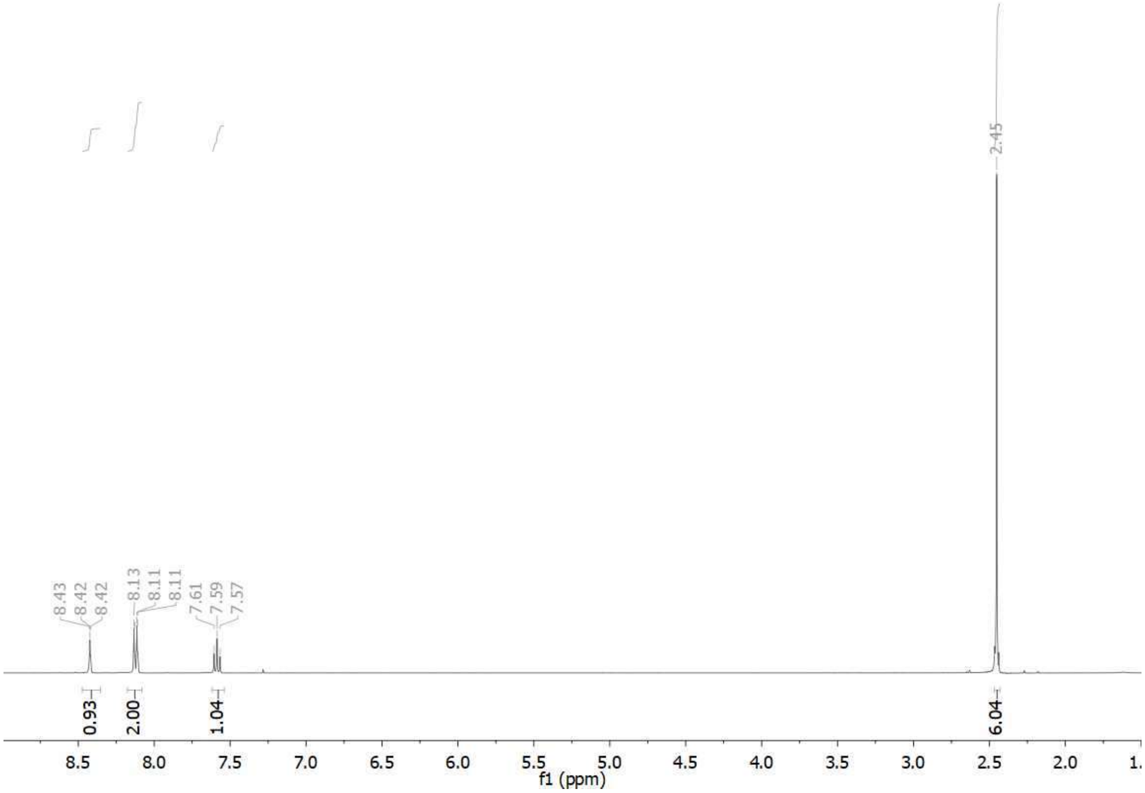

Figure S2. ^{1}H-NMR of compound 4 in $CDCl_3$.

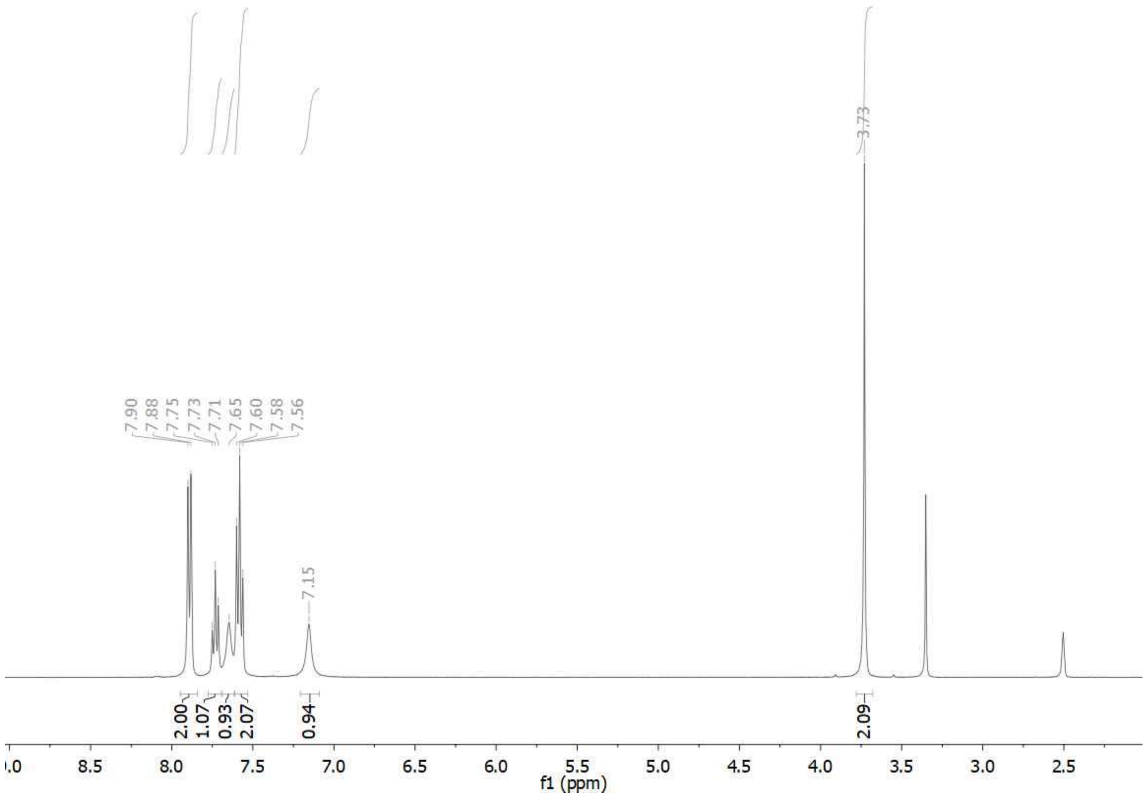

Figure S3. ^{1}H-NMR of compound 6 in DMSO-d_6.

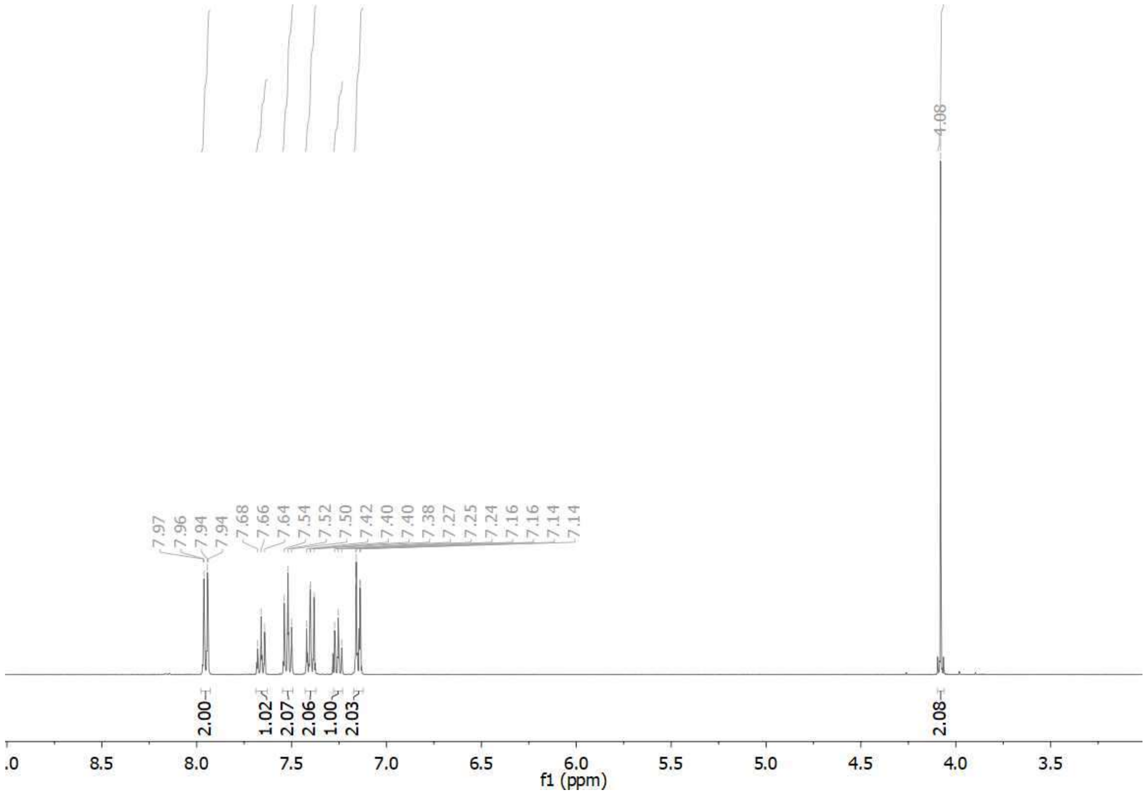

Figure S4. ^{1}H-NMR of compound 8 in $CDCl_3$.

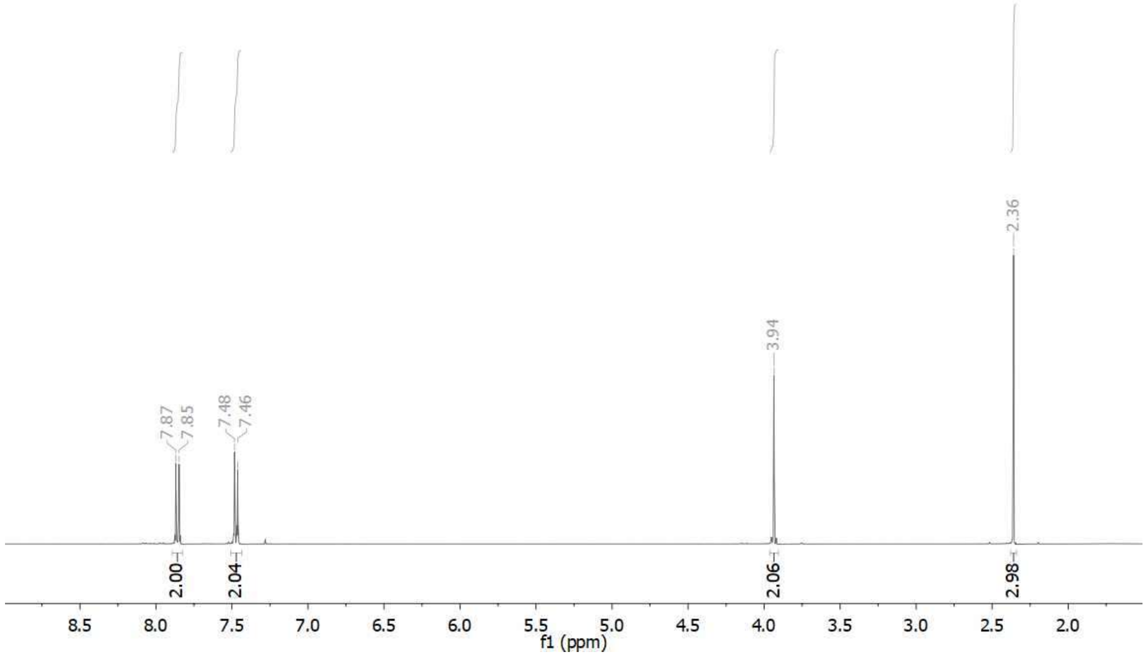

Figure S5. ^{1}H-NMR of compound 9 in $CDCl_3$.

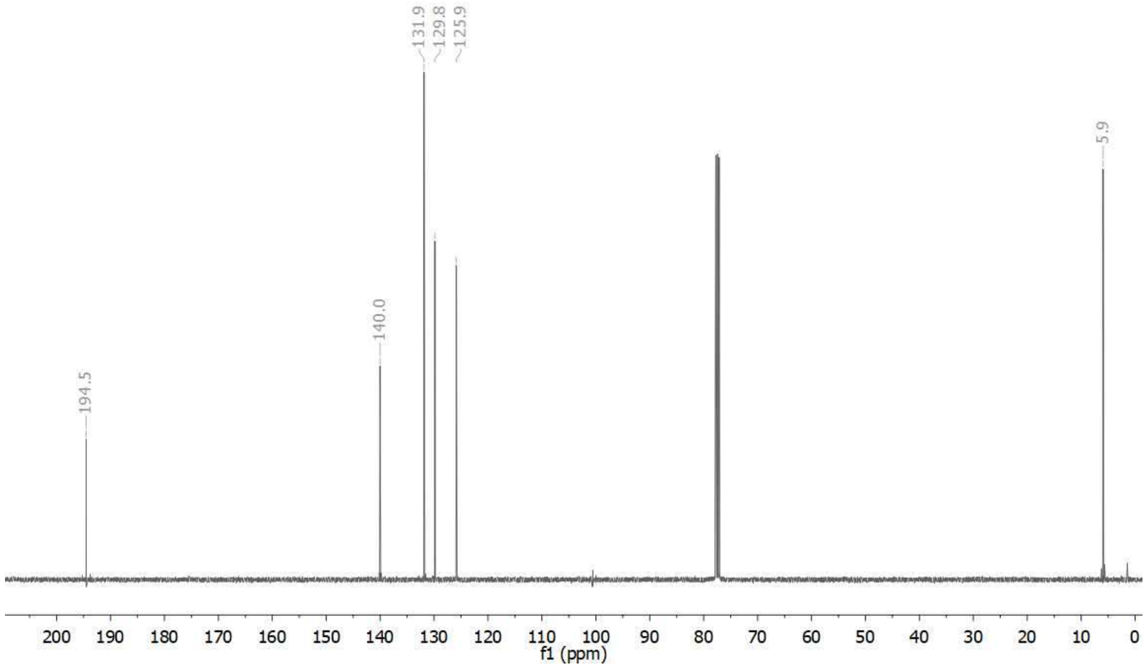

Figure S6. ^{13}C-NMR of compound 4 in $CDCl_3$.

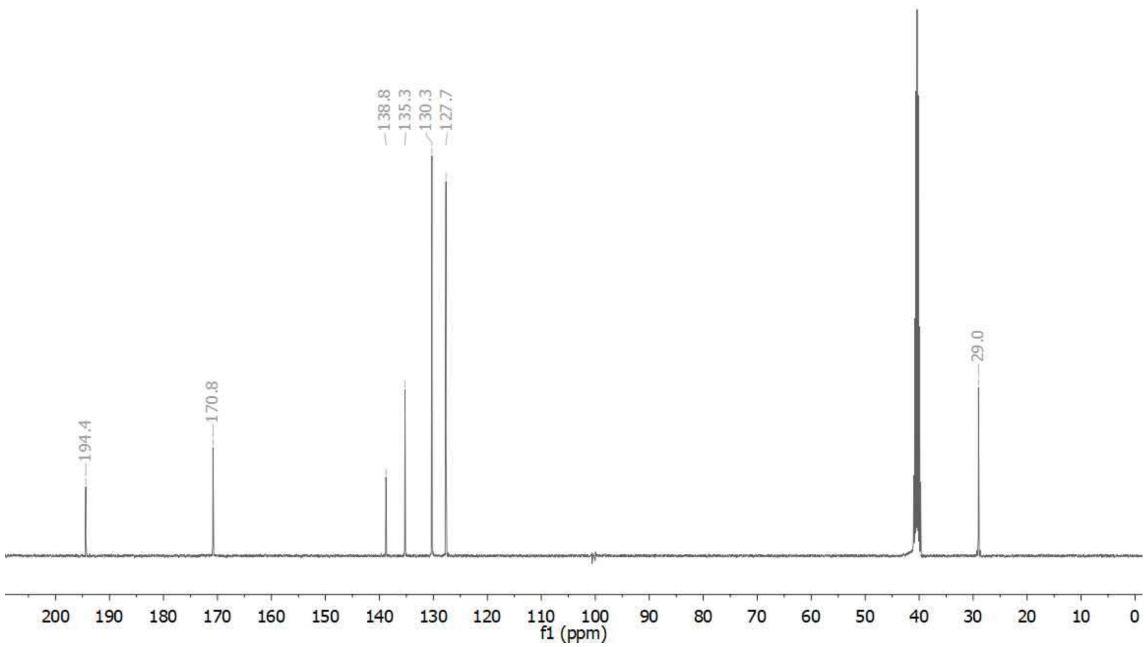

Figure S7. ^{13}C-NMR of compound 6 in DMSO-d_6.

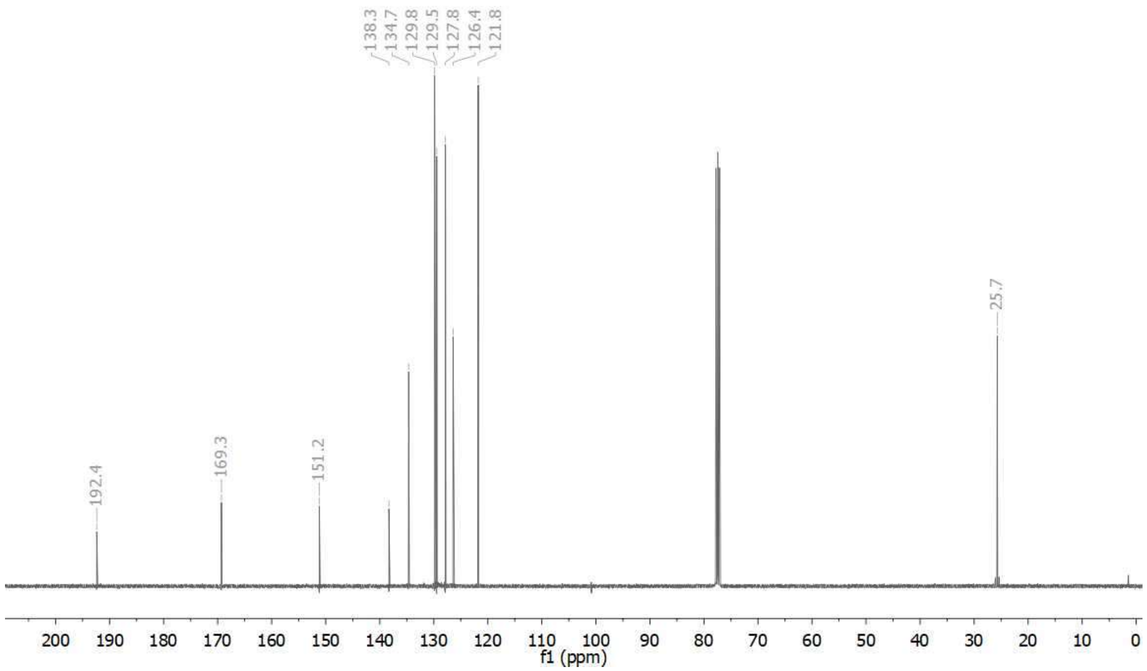

Figure S8. ^{13}C-NMR of compound 8 in $CDCl_3$.

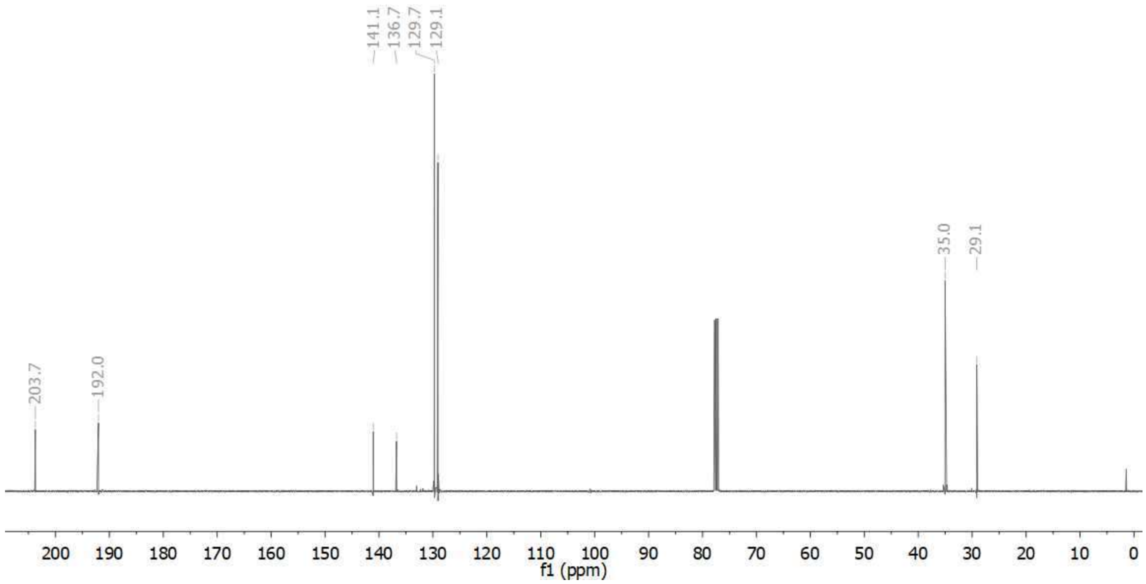

Figure S9. ^{13}C-NMR of compound 9 in $CDCl_3$.
